# BI-847325, a selective dual MEK and Aurora kinases inhibitor, reduces aggressive behavior of anaplastic thyroid carcinoma on an in vitro three-dimensional culture

**DOI:** 10.1186/s12935-022-02813-6

**Published:** 2022-12-08

**Authors:** Hilda Samimi, Rezvan Tavakoli, Parviz Fallah, Alireza Naderi Sohi, Maryam Amini Shirkouhi, Mahmood Naderi, Vahid Haghpanah

**Affiliations:** 1 Endocrinology and Metabolism Research Center, Endocrinology and Metabolism Clinical Sciences Institute, Tehran University of Medical Sciences, Tehran, Iran; 2 Department of Biology, Science and Research Branch, Islamic Azad University, Tehran, Iran; 3 Hepatitis and HIV Department, Pasteur Institute of Iran, Tehran, Iran; 4 Department of Laboratory Science, Faculty of Allied Medicine, Alborz University of Medical Sciences, Karaj, Iran; 5 Digestive Diseases Research Center, Digestive Diseases Research Institute, Tehran University of Medical Sciences, Tehran, Iran; 6 Personalized Medicine Research Center, Endocrinology and Metabolism Clinical Sciences Institute, Tehran University of Medical Sciences, Tehran, Iran

**Keywords:** Anaplastic thyroid carcinoma, BI-847325, MEK, Aurora kinase

## Abstract

**Background:**

Anaplastic thyroid carcinoma (ATC) is the most aggressive subtype of thyroid cancer. In this study, we used a three-dimensional in vitro system to evaluate the effect of a dual MEK/Aurora kinase inhibitor, BI-847325 anticancer drug, on several cellular and molecular processes involved in cancer progression.

**Methods:**

Human ATC cell lines, C643 and SW1736, were grown in alginate hydrogel and treated with IC_50_ values of BI-847325. The effect of BI-847325 on inhibition of kinases function of MEK1/2 and Aurora kinase B (AURKB) was evaluated via Western blot analysis of phospho-ERK1/2 and phospho-Histone H3 levels. Sodium/iodide symporter (*NIS*) and thyroglobulin (*Tg*), as two thyroid-specific differentiation markers, were measured by qRT-PCR as well as flow cytometry and immunoradiometric assay. Apoptosis was assessed by Annexin V/PI flow cytometry and *BIM*, *NFκB1*, and *NFκB2* expressions. Cell cycle distribution and proliferation were determined via *P16, AURKA, and AURKB* expressions as well as PI and CFSE flow cytometry assays. Multidrug resistance was evaluated by examining the expression of *MDR1* and *MRP1*. Angiogenesis and invasion were investigated by *VEGF* expression and F-actin labeling with Alexa Fluor 549 Phalloidin.

**Results:**

Western blot results showed that BI-847325 inhibits MEK1/2 and AURKB functions by decreasing phospho-ERK1/2 and phospho-Histone H3 levels. BI-847325 induced thyroid differentiation markers and apoptosis in ATC cell lines. Inversely, BI-847325 intervention decreased multidrug resistance, cell cycle progression, proliferation, angiogenesis, and invasion at the molecular and/or cellular levels.

**Conclusion:**

The results of the present study suggest that BI-857,325 might be an effective multi-targeted anticancer drug for ATC treatment.

**Supplementary Information:**

The online version contains supplementary material available at 10.1186/s12935-022-02813-6.

## Background

Anaplastic thyroid carcinoma (ATC) is one of the most lethal malignancies, with a median survival rate of six months. In general, surgical- and radioiodine-based therapeutic procedures are not effective, and ATC patients have poor prognoses with high morbidity and mortality. At present, there is no standard therapy for ATC, and the patientsʼ survival rate has not improved for decades [1, 2].

Considerable progress has been made in identifying genetic changes and signaling pathways, involved in the invasiveness of ATC. Several new therapies that target the relevant pathways are currently undergoing clinical trials [1]. In ATC, most genetic mutations, importantly *BRAF*^V600E^, affect the MEK/ERK signaling pathway [3]. Activation of the Mitogen-Activated Protein Kinase (MAPK) Pathway plays a key role in the regulation of the cell cycle, apoptosis, proliferation, differentiation, invasion, angiogenesis, and drug resistance [4, 5]. At present, genetic-based targeted therapy is the most promising curative plan in ATC. A wide range of inhibitors have been discovered against the main effectors of the MAPK pathway that demonstrate anticancer potential by suppressing the growth of tumor cells both in vitro and in vivo [1]. Since MEK is the downstream effector of BRAF, MEK inhibition is an effective strategy to block the reactivation of the MAPK pathway in ATC. In 2018, Dabrafenib, a BRAF inhibitor, in combination with Trametinib, a MEK inhibitor, were approved by the Food and Drug Administration (FDA) for the treatment of ATC patients with the *BRAF*^V600E^ mutation [6, 7].

In addition to genetic alterations, the ability of high proliferation, a hallmark of cancers, is thought to represent a major driving force in ATC progression and poor prognosis [8]. A number of mitotic kinases with irregular expression in malignant thyroid tissues are involved in the growth of thyroid tumors. These include three members of the Aurora kinase family (AURKA, AURKB, and AURKC), serin/thereonine kinases that regulate multiple aspects of tumor growth and progression [9]. In recent years, a wide range of Aurora kinase inhibitors have been developed that have shown promising antitumor effects against ATC in preclinical studies [10].

At present, several hybrid compounds are introduced for cancer treatment that are multi-target in nature due to simultaneously interfering with different signaling pathways involved in cancer development [11, 12]. BI-847325, a selective dual MEK/Aurora kinase inhibitor, is one of the multi-target anticancer drugs [13]. This hybrid compound may reduce the risk of adverse drug reactions and side effects [13–15]. However, despite the acceptable safety profile of BI-847325, its development has been halted due to insufficient drug exposure at the maximum tolerated dose (MTD) to achieve MEK inhibition in cancers such as liver, kidney, breast, and melanoma [16].

The microenvironment is an emerging concept in cancer biology that is shown to dramatically alter not only the behavior of tumor cells but also drug responses. However, most in vitro studies testing drug effects on cancer cells use two-dimensional (2D) cell culture protocols. In a traditional 2D culture system, adherent tumor cells are attached to the plastic surface of a Cell Culture Flask or Flat Petri Dish and grown as a monolayer. This routine method of cell culture has advantages such as simple maintenance and cost-effectiveness, but it also has the disadvantage of incompetence in simulating the tumor microenvironment [17]. Indeed, three-dimensional (3D) cell culture models better mimic the natural tumor microenvironment such as physiological gradient, cell-cell communications, and cell-matrix interactions. Therefore, 3D cell culture systems have an important role in the discovery of anticancer drugs. In recent years, the use of 3D models in cancer research has substantially increased [18, 19].

In the present study, the effect of dual MEK/Aurora kinase inhibitor BI-847325 on the key biological processes involved in cancer progression including cell cycle, apoptosis, proliferation, differentiation, invasion, angiogenesis, and drug resistance was evaluated using a 3D in vitro ATC model at the molecular and/or cellular levels.

## Materials and methods

### 2D cell culture

Human ATC cell lines, C643 and SW1736, were purchased from Cell Lines Service (CLS, Germany). Both of the cell lines were cultured according to the providerʼs instruction.

### 3D cell culture and cytotoxicity assay

C643 and SW1736 ATC cell lines were grown in the alginate hydrogel [20, 21]. The cytotoxic effect of BI-847325 (Adooq Bioscience, Irvine, CA, USA, Cat. No.: A15762) on 3D-cultured cells was measured in 24, 48, and 72 h by MTT [3-(4,5-dimethylthiazolyl-2)-2,5-diphenyltetrazolium bromide] assay. The half-maximal inhibitory concentration (IC_50_) of BI-847325 anticancer drug was calculated in 3D cell culture system for each ATC cell line as previously described [20, 21].

### Cell cycle assay

The spheroids were pre-cultured before BI-847325 treatment. Then, C643 and SW1736 cell lines were treated with 15 µM and 34 µM concentrations (IC_50_ values) of BI-847325 for 48 h, respectively. Cells were revived from the alginate scaffold using sodium citrate (55 mM, 10 mM HEPES, pH 7.4) as previously explained [22] and analyzed through the Cell Cycle Assay Kit (Abcam, Cambridge, USA) according to the manufactureʼs protocol. Cell cycle distribution for each cell line was evaluated by flow cytometry using BD FACSCalibur. The percentage of cells in the G0/G1, S, and G2/M phases was determined using FlowJo software.

### Apoptosis assay

Treated cells with IC_50_ values of BI-847325 were revived from the hydrogel scaffold. For apoptosis assay, the Annexin V-FITC Apoptosis Detection Kit (Invitrogen, Carlsbad, CA, USA) was used. The apoptotic cells were detected and quantified by flow cytometry using BD FACSCalibur. The data was analyzed using FlowJo software.

### Cell proliferation assay

Before alginate encapsulation and treatment, cells were labeled using CellTrace™ CFSE Cell Proliferation Kit (Invitrogen, Carlsbad, CA, USA) according to the manufactureʼs instruction. The proliferation capacity of C643 and SW1736 was measured by flow cytometry using BD FACSCalibur after treatment with IC_50_ values of BI-847325 and decapsulation. The proliferation data were analyzed using FlowJo software.

### Flow cytometric detection of sodium/iodide symporter (NIS)

The cell lines were treated with IC_50_ values of BI-847325 and removed from the scaffold after 48 h. The amount of NIS protein was assessed using the anti-human NIS antibody (Abcam, Cambridge, UK, Cat. No.: ab17795) by flow cytometry according to the manufactureʼs protocol. Isotype control antibody (mouse IgG1, Abcam, Cambridge, UK) was used under the same condition. The relative expression of NIS protein was determined using FlowJo software by comparing the mean fluorescence intensity between treated and untreated cells.

### Immunoradiometric assay (IRMA)

Culture condition, treatment, and decapsulation have been described above. Before h-thyroglobulin (Tg) measurement, cell lines were physically lyzed by sonication with 15-W power for 30 s with consecutive 1 s ON and 1 s OFF power (Bandelin, model HD3100). The amount of Tg protein was measured by IRMA Kit (Isotopes Co., Ltd. (IZOTOP), Budapest, Hungary), based on the manufactureʼs guideline.

### Invasion assay

Invasion of pre- and post-treatment of C643 and SW1736 ATC cell lines was evaluated following fixation of alginate-encapsulated cells with 4% paraformaldehyde. The nuclei and actin filaments were stained with DAPI and Alexa Fluor™ 549 Phalloidin (Invitrogen, Carlsbad, CA, USA), respectively. Then, the stained cells were imaged by the fluorescence microscopy.

### Western blotting assay

After decapsulating the cells from the hydrogel, protein extraction and Western blotting were carried out as described by Eslami et al. [23]. The following primary antibodies were used to confirm the inhibitory effect of BI-847325 on the function of MEK1/2 and AURKB kinases in the treated cell lines: Total p44/42 MAPK (ERK1/2), Phosphorylated p44/42 MAPK (ERK1/2)^Thr202/Tyr204^, Histone H3, Phospho-Histone H3 (Ser10) (Cell Signaling Technologies, UK, Cat. No.: #9102, #4370, #4499, #53,348, respectively), and β-actin (PADZA, Tehran, Iran, Cat. No.: MM114). Secondary antibodies included HRP-conjugated goat anti-rabbit IgG and HRP-conjugated goat anti-mouse IgG were a kind gift from Dr. Mohammad Reza Nejadmoghaddam (Avicenna Research Institute, Tehran, Iran).

### Quantitative real-time PCR (qRT-PCR) assay

Treated cells with IC_50_ values of BI-847325 were removed from the alginate scaffold after 48 h. RNA isolation, complementary DNA (cDNA) synthesis, and qRT-PCR were performed as previously described [22]. Changes in mRNA expression were normalized to β-actin. The primer sequences are listed in Table 1.

**Table 1:** The sequences of the primers

Gene	Sequence
*AURKA*	F: ATCGGCACCTGAAAATAATCC R: TCTTCCAAAGCCCACTGC
*AURKB*	F: CATCTGCACTTGTCCTCATG R: GAAGTGCCGCGTTAAGATG
*BIM*	F: CACTACCACCACTTGATTCTTG R: GGTCACACTCAGAACTTACATC
*MDR1*	F: TGGACAAGCACTGAAAGATAAG R: TTCCTCAAAGAGTTTCTGTATGG
*MRP1*	F: CCATCCACGACCCTAATCC R: CGCATTCCTTCTTCCAGTTC
*NFκB2*	F: GAGTTGCTACAACCCAGGTC R: ACAGTGGGATAGGTCTTTCG
*P16*	F: GAAGGTCCCTCAGACATCC R: AATGGACATTTACGGTAGTGG
*Tg*	F: GGTTCCTCGCAGTTCAATC R: GCCTTCAGCACAAGATGG
*VEGF*	F: ATCACGAAGTGGTGAAGTTC R: TGAGGTTTGATCCGCATAATC
*NIS*	F: CATCCTGAACCAAGTGACC R: TAGCATCACCACGACCTG
*NFκB1*	F: GTGCTGGAGTTCAGGATAACC R: GTGGATGATTGCTAAGTGTAA GAC
*β-actin*	F: CTTCCTTCCTGGGCATG R: GTCTTTGCGGATGTCCAC

*AURKA* Aurora Kinase A, *AURKB* Aurora Kinase B, *AURKBC* Aurora Kinase C, *BIM* BCL2 Like 11, *MDR1* ATP Binding Cassette Subfamily B Member 1, *MRP1* ATP Binding Cassette Subfamily C Member 1, *NFκB1* Nuclear Factor Kappa B Subunit 1, *NFκB2* Nuclear Factor Kappa B Subunit 2, *NIS* Sodium/Iodide Symporter, *P16* Cyclin Dependent Kinase Inhibitor 2 A, *p-H3* phospho-Histone H3, *TFs* Transcription Factors, *Tg* Thyroglobulin, *VEGF* Vascular Endothelial Growth Factor

### Statistical analysis

All the experiments except Western blot were performed two times. Data were analyzed using GraphPad Prism software (GraphPad PRISM V 5.0) and presented as means ± standard deviation (SD). The gene expression analyses were done using 2^–∆∆Ct^ method. Two-way ANOVA was used for the statistical analysis of the cell cycle and apoptosis experiments. The statistical significance of other assays was determined by *t*-test. Statistical significances are expressed as *p* < .05 (*); *p* < .01 (**); *p* < .001 (***); *p* < .0001 (****).

## Results

### qRT-PCR

#### BI-847325 regulated cell cycle and proliferation through upregulation of *P16* and downregulation of *AURKA* and *AURKB*

**Fig. 1:**
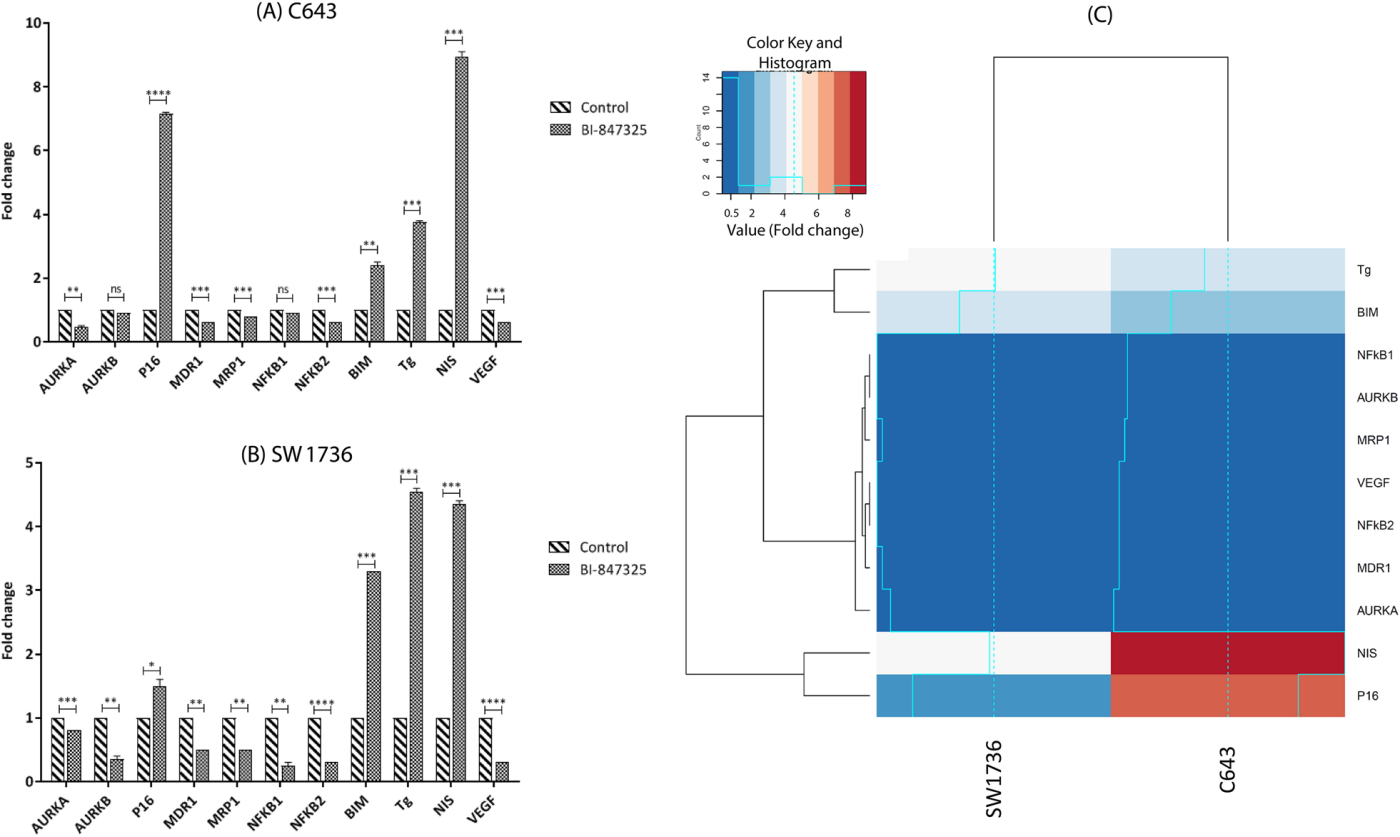
The effect of BI-847325 on the expression of different inspected genes in C643 and SW1736 ATC cell lines. **A** The expression of *AURKA*, *MDR1*, *MRP1*, *NFκB2*, and *VEGF* genes was significantly decreased in C643 cell line. The expression of *P16* and *BIM* as well as *NIS* and *Tg* genes was significantly increased in this cell line. All the expressions were compared to the untreated control 48 h after treatment with 15 µM of BI-847325. **B** The expression of *AURKA*, *AURKB*, *MDR1*, *MRP1*, *NFκB1, NFκB2*, and *VEGF* genes was significantly decreased in SW1736 cell line. The expression of *P16* and *BIM* as well as *NIS* and *Tg* genes was significantly increased in this cell line. All the expressions were compared to the untreated control 48 h after treatment with 34 µM of BI-847325. Statistical significances are expressed as *p* < .05 (*); *p* < .01 (**); *p* < .001 (***); *p* < .0001 (****). **C** Gplot heatmap of gene expression data in C643 and SW1736 ATC cell lines. The differences between the gene expression profile of *AURKA*, *AURKB*, *MDR1*, *MRP1*, *NFκB1, NFκB2*, *VEGF*, *P16*, *BIM*, *NIS*, and *Tg* in C643 and SW1736 are shown as color-coded: crimson for upregulated and dark blue for downregulated genes

The expression of *P16*, as a suppressor of cell cycle progression at the G1/S checkpoint [24], was measured after BI-847325 treatment on C643 and SW1736 cell lines. As shown in Fig. 1A and B, the expression of *P16* was significantly upregulated after treatment in both ATC cell lines. Also, the expression of key regulators of the G2/M transition of the mitotic cell cycle, *AURKA* [25] and *AURKB* [26], were evaluated following treatment. The data showed that the expression of *AURKA* was significantly decreased in comparison with untreated control in C643 and SW1736. However, the expression of *AURKB* was merely downregulated SW1736 cell line (Fig. 1A and B).

#### BI-847325 induced apoptosis and necrosis via upregulation of *BIM* and downregulation of *NFκB1* and *NFκB2*

Inhibition of MEK activity was shown to regulate the expression of pro-apoptotic gene *BIM* and anti-apoptotic genes *NFκB1* and *NFκB2*. As shown in Fig. 1A and B, the expression of *BIM* was significantly upregulated by BI-847325 treatment in both ATC cell lines. Reversely, the expression of *NFκB2* was significantly decreased following treatment in the two cell lines. However, the expression of *NFκB1* was significantly downregulated by BI-847325 just in SW1736 cell line. It should be noted that the expression of *AURKA* and *AURKB* genes, which are also involved in inducing apoptosis, decreased after treatment as mentioned above.

#### BI-847325 decreased multidrug resistance through downregulation of *MDR1* and *MRP1*

Suppression of MAPK signaling pathway activity by BI-847325 treatment could significantly decrease the expression of *MDR1* and *MRP1* genes in C643 and SW1736 ATC cell lines (Fig. 1A and B).

#### BI-847325 induced redifferentiation via upregulation of *NIS* and *Tg*

To evaluate the amount of differentiation in C643 and SW1736 cells following drug intervention, the expressions of *NIS* and *Tg*, as two thyroid-specific differentiation markers, were measured. The qRT-PCR results showed that the expressions of both genes were significantly increased in BI-847325 treated cells in comparison with the control (Fig. 1A and B).

#### BI-847325 decreased angiogenesis through downregulation of *VEGF*

Inhibition of the MEK/ERK pathway was shown to decrease the expression of *VEGF* gene. As shown in Fig. 1A and B, the expression of *VEGF* was significantly downregulated following BI-847325 treatment in C643 and SW1736 ATC cell lines.

The differences in the gene expression patterns of all measured genes in C643 and SW1736 cells are shown in Fig. 1C.

### Flow cytometry and IRMA

#### BI-847325 regulated cell cycle

**Fig. 2:**
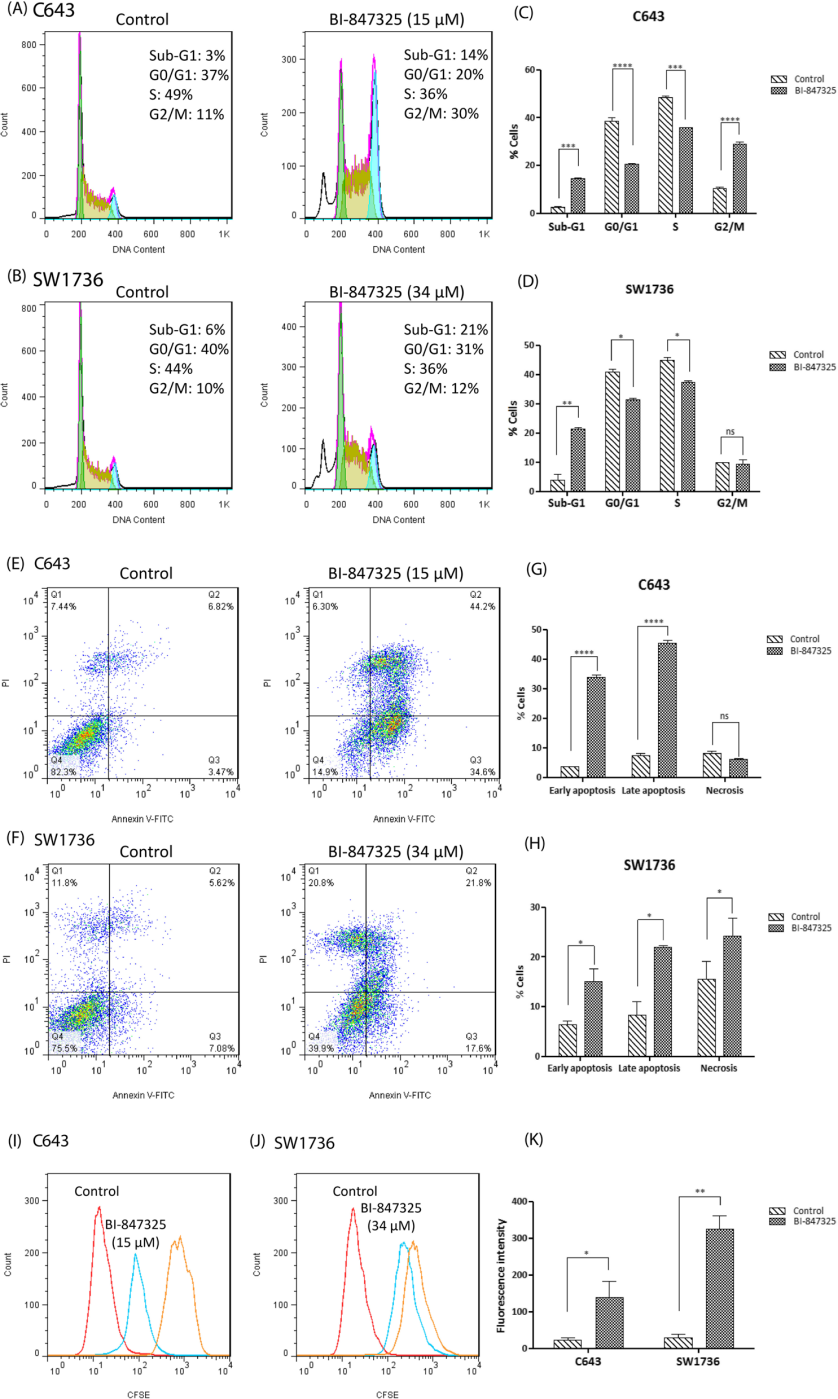
The effect of BI-847325 on the cell cycle distribution, apoptosis, and proliferation in C643 and SW1736 ATC cell lines. **A, B** After 48 h of drug intervention, C643 and SW1736 cells were subjected to cell cycle analysis by PI flow cytometry. The percentage of cells in sub-G1, G0/G1, S, and G2/M phases are indicated for each cell line. The untreated cells were considered as control. **C, D** Representative histogram of cell cycle analysis based on flow cytometry in 48 h after BI-847325 treatment of C643 and SW1736 compared to untreated cells as control. **E, F** Apoptosis assessment using Annexin V/PI flow cytometry in BI-847325 treated cells in comparison with untreated cells as control. **G, H** Graphical representation of early apoptosis, late apoptosis, and necrosis based on flow cytometry in 48 h after BI-847325 treatment of C643 and SW1736 compared to untreated cells as control. Cellular proliferation of untreated and treated cells was measured using flow cytometry 48 h after treatment. **I, J** CFSE histograms for C643 and SW1736. Orange histograms show 18 h after labeling, which confirms labeled but non-proliferating cells. **K** The diagram shows significantly higher fluorescence intensity and consequently lower proliferation in treated ATC cell lines compared to untreated cells. Statistical significances are expressed as *p* < .05 (*); *p* < .01 (**); *p* < .001 (***); *p* < .0001 (****)

Changes in the cell cycle distribution induced after BI-847325 treatment were evaluated by cellular DNA content and cell cycle profiling using flow cytometry. The treatment of ATC cells caused different results in cell cycle phase distribution in each cell line (Fig. 2A–D). BI-847325 decreased the number of cells in the G0/G1 and S phases in C643 and SW1736 cells and increased the accumulation of cells in G2/M just in C643 cell line. Moreover, as shown in Fig. 2A–D, BI-847325 significantly induced apoptosis, which was measured by the proportion of sub-G1 cells compared to the controls. Flow cytometry analysis further confirmed the related data in mRNA levels of *P16*, *AURKA*, and *AURKB* genes concerning the effect of BI-843,725 anticancer drug on the cell cycle regulation.

#### BI-847325 induced apoptosis

The inhibition of the MAPK signaling pathway and the members of Aurora kinase family may lead to the induction of apoptosis. The ability of BI-847325 to cause apoptotic/necrotic cell death in ATC cell lines was analyzed using flow cytometry. Treatment of C643 and SW1736 caused different results in each cell line. As shown in Fig. 2E–H, BI-847325 treatment resulted in significant induction of death in two ATC cell lines, 67% in C643 and 35.7% in SW1736. Flow cytometry analysis of cellular apoptosis further confirmed the obtained data concerning the effect of BI-847325 on cell death via transcript-level regulation of apoptotic-/necrotic-associated genes including *BIM*, *NFκB1*, *NFκB2*, *AURKA*, and *AURKB* as well as the increased number of cells in sub-G1.

#### BI-847325 decreased cell proliferation

MEK/ERK signaling pathway and Aurora kinase family play an important role in the regulation of ATC cell proliferation. Therefore, flow cytometry assay was performed to determine the effect of dual MEK/Aurora kinase inhibitor BI-847325 on cell proliferation. As shown in Fig. 2I–K, the treatment with BI-847325 resulted in a significant reduction of cell proliferation in C643 and SW1736 cell lines. Moreover, as mentioned above, changes in the expression of proliferation-related genes were consistent with the results at the cellular level.

#### BI-847325 induced cell redifferentiation

**Fig. 3:**
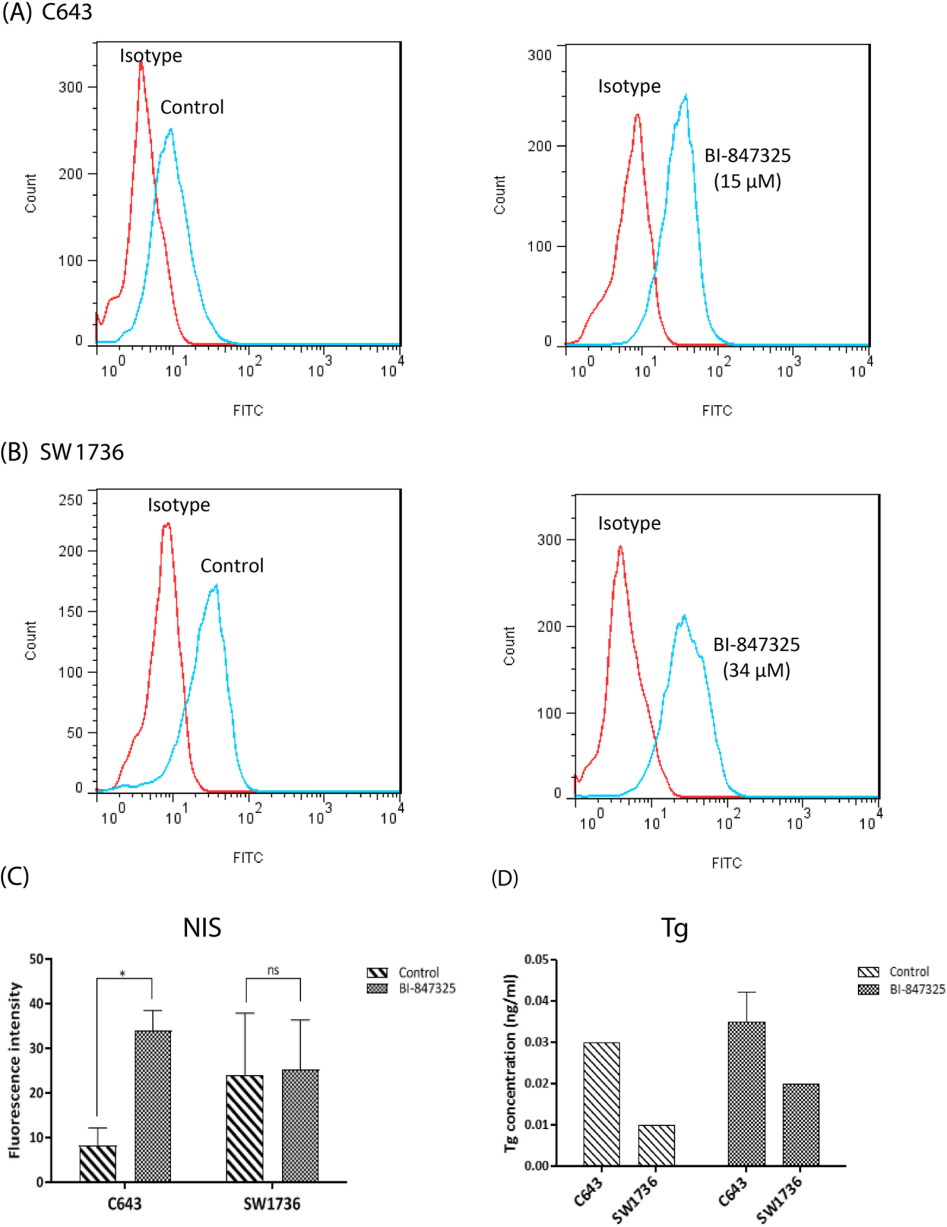
The effect of BI-847325 on the protein levels of NIS and Tg in C643 and SW1736 ATC cell lines. NIS was quantified by flow cytometry 48 h after BI-847325 treatment. The Representative histograms indicate the FITC unspecific background fluorescence (isotype) and the level of NIS protein Pre- and post-treatment in C643 **A** and SW1736 **B**. **C** Graphical representation of the protein level of NIS based on flow cytometry in 48 h after BI-847325 treatment of ATC cell lines compared to untreated cells as control. Tg was measured using IRMA assay 48 h after BI-847325 treatment. **D** The concentration of Tg protein is indicated for C643 and SW1736 cells. Statistical significances are expressed as *p* < .05 (*); *p* < .01 (**); *p* < .001 (***); *p* < .0001 (****)

To investigate whether BI-847325 could influence the differentiation of ATC cells, the expressions of *NIS* and *Tg* genes were measured by qRT-PCR. Furthermore, flow cytometry assay and IRMA were used to evaluate the protein levels of NIS and Tg, respectively. Inhibition of MAPK pathway by BI-847325 significantly enhanced transcriptomic levels of *NIS* and *Tg* in C643 and SW1736 cell lines, as described above. Moreover, flow cytometry results showed that the NIS protein was significantly upregulated by BI-847325 treatment in C643 cell line. However, the protein level of NIS was not significantly increased after treatment in SW1736 cells (Fig. 3A–C). Also, IRMA results were further confirmed the elevated level of *Tg* in the treated cells in comparison with the untreated control in both cell lines, although this increase was not significant (Fig. 3D). This may be related to the difference in the expression level of *NIS* after treatment in the two cell lines (Fig. 1C). The different observations at the cellular and molecular levels in each of the cell lines give us a wide view concerning the genetic background difference of C643 and SW1736 ATC cell lines [27–30].

### F-actin labeling

#### BI-847325 decreased invasion by reducing F-actin network formation

**Fig. 4:**
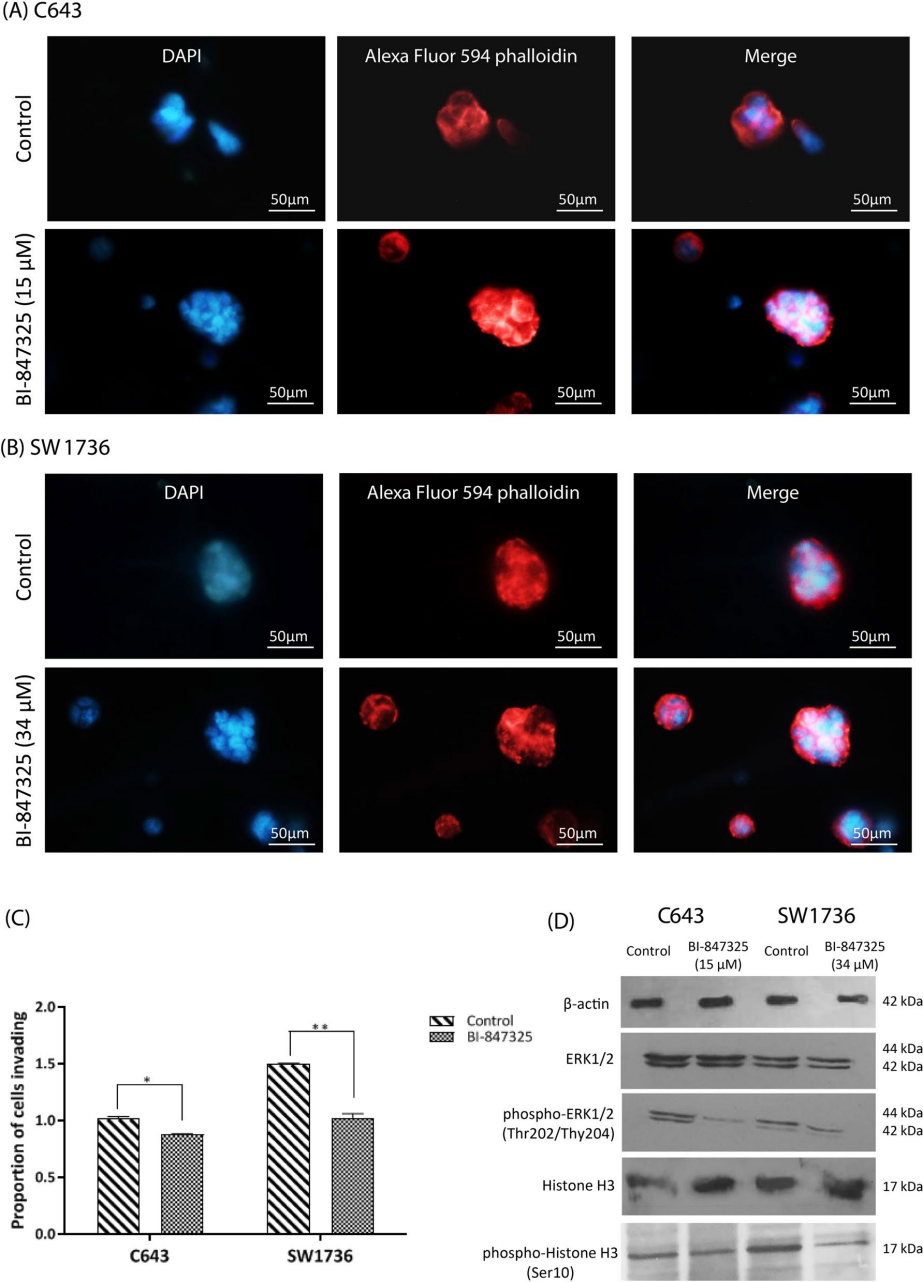
The effect of BI-847325 on invasion of ATC cell lines as well as inhibition of the kinases function of MEK1/2 and AURKB. Alexa Fluor 549 Phalloidin staining was performed to investigate changes in the invasiveness of C643 **A** and SW1736 **B** 48 h after BI-847325 treatment. The nuclei were also stained simultaneously with DAPI (A-B, magnification = 400X, Scale bar = 50 μm). **C** The proportion of cell invading is indicated for C643 and SW1736. Statistical significances are expressed as *p* < .05 (*); *p* < .01 (**); *p* < .001 (***); *p* < .0001 (****). **D** This hybrid compound decreased the function of MEK1/2 and AURKB by reducing the levels of phospho-ERK1/2 and phospho-Histone H3 in C643 and SW1736 cell lines. Uncropped immunoblots are shown in Additional file 1: Fig. S1

To assess the effect of BI-847325 on the invasiveness of ATC cells, F-actin labeling was used to determine BI-847325-induced actin cytoskeleton remodeling. The result showed that the formation of F-actin network in cell invasion was significantly decreased following inhibition of the MEK/ERK pathway and Aurora kinase family in C643 and SW1736 cell lines (Fig. 4A–C).

### Western blotting

#### BI-847325 inhibited the kinases function of MEK1/2 and AURKB

The phosphorylated form of ERK1/2 and Histone H3 are upregulated in ATC and contribute to the highly aggressive behavior of this tumor. The effect of BI-847325 on inhibition of kinases function of MEK1/2 and AURKB in ATC cell lines was examined by Western blot assay. The results confirmed that the treatment with BI-847325 decreased the levels of phospho-ERK1/2 and phospho-Histone H3, without altering protein expression (Fig. 4D).

## Discussion

**Fig. 5:**
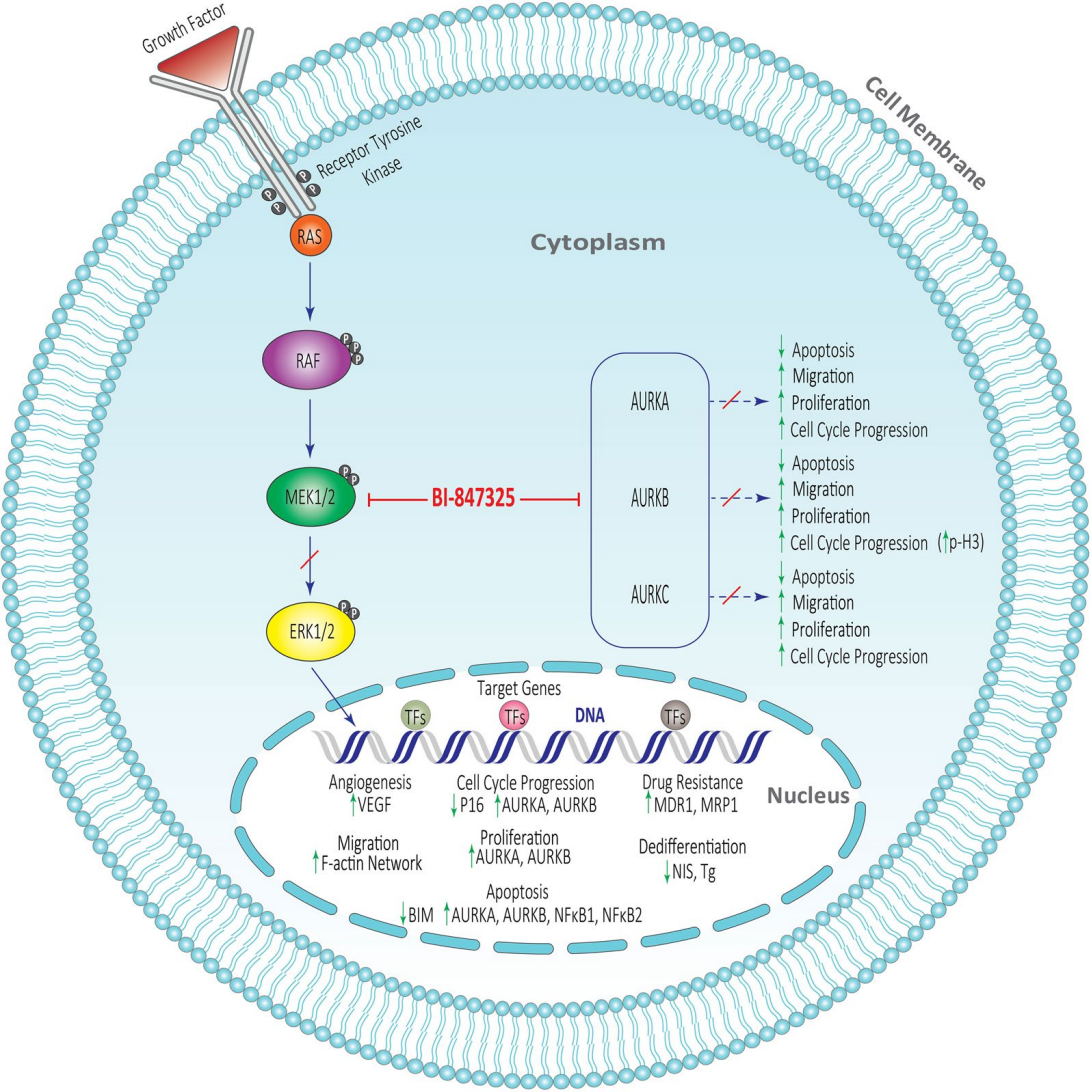
The effect of BI-847325, as a selective dual MEK and Aurora kinases inhibitor, on the fundamental cellular and molecular mechanisms including cell cycle, apoptosis, proliferation, invasion, angiogenesis, drug resistance, and differentiation in ATC cells

In this study, to evaluate the anticancer effect of BI-847325 on ATC, various biological processes associated with cancer progression including drug resistance, differentiation, angiogenesis, invasion, apoptosis, cell cycle, and proliferation were examined at the molecular and/or cellular levels (Fig. 5).

Currently, a multimodal therapeutic approach including surgery followed by chemotherapy is used for ATC patients. Unfortunately, genotoxic drugs such as Doxorubicin are not very efficient against ATC. Therefore, molecular targeted drugs including tyrosine kinase inhibitors are recommended for more effective treatment of patients with ATC. However, the prognosis of patients is poor and mortality from this cancer remains a major challenge worldwide [1]. Chemotherapy studies have shown that Doxorubicin, as single-agent or in combination, produces response rates between 15% and 25% [31, 32]. Several mechanisms have been proposed in relation to Doxorubicin drug resistance. One of the most important of them is the overexpression of ATP-binding cassette transporters such as MDR1 and MRP1 [33]. Studies have shown that the expression of these multidrug resistance genes can be regulated by the MEK/ERK signaling pathway in ATC [34]. As the results of this study show, the treatment with targeted anticancer drug BI-847325 could reduce the expression of multidrug resistance genes, *MDR1* and *MRP1*, in ATC cell lines (Fig. 1). Hence, it is expected that the modified cells with reduced drug resistance capacity are more sensitive to anticancer drugs. However, further studies at the cellular and tumoral levels are needed to confirm this molecular finding.

As evidence has shown, during the development of ATC, thyroid cells lose their differentiation and consequently the expression of NIS and Tg, as two differentiation markers of thyrocytes. Therefore, ATC patients do not respond to radioiodine ablation as the mainstay of treatment for differentiated thyroid cancer, due to decreased expression of NIS [35–38]. Any drug intervention that leads to the expression of NIS can be considered as a candidate for chemotherapy of ATC by inducing redifferentiation and providing the basis for treatment with radioiodine [39–41]. Therefore, NIS and Tg, whose expression is regulated by the MAPK pathway [42], were evaluated to assess the degree of differentiation induced in ATC cells by BI-847325. BI-847325 had the potential to increase the expression of NIS and Tg at the transcript and protein levels and may produce cells that are more sensitive to radioactive iodine therapy (Figs. 1 and 3). Therefore, it can be considered as an effective multi-targeted anticancer drug for the treatment of ATC and other radioiodine-refractory thyroid cancers.

Angiogenesis and actin cytoskeleton organization also play an important role in tumor progression and invasion of ATC [43, 44]. Angiogenesis is affected by vascular endothelial growth factor (VEGF), and studies have shown that the expression of *VEGF* gene is upregulated in thyroid cancer, especially in ATC. Tyrosine kinase inhibitors of VEGF receptors are currently used as targeted therapies for ATC patients [43, 45, 46]. The treatment of ATC cell lines with BI-847325 may decrease tumor angiogenesis by reducing *VEGF* expression (Fig. 1). Han et al. demonstrated that the MAPK pathway regulates actin organization and cell invasion [47]. In addition, research has shown that three members of the Aurora kinase family (AURKA, AURKB, and AURKC) play an important role in cancer cell migration and tumor progression [48–50]. Simultaneous inhibition of the MEK/ERK pathway and Aurora kinase family by BI-847325 remodeled the actin filament network and reduced ATC cell invasion (Fig. 4 A-C). However, further studies are needed to determine the role of Aurora kinases in the organization of actin filaments.

As mentioned, MAPK signaling is one of the most important intracellular pathways involved in ATC development and progression [51]. This signaling pathway reduces cell death by altering the expression of genes involved in the apoptotic process such as *BIM*, *NFκB1*, *NFκB2*, *AURKA*, and *AURKB*, thereby increasing tumor growth [52–55]. Also, studies have shown that the Aurora kinase family is involved in tumor progression by suppressing apoptosis [56, 57]. Selective inhibitors of AURKA, AURKB, and AURKC can induce apoptosis in ATC cells [58, 59]. For instance, Bonnemains et al. indicated that VX-680 increased apoptosis by inhibiting the function of AURKA, AURKB, and AURKC in different ATC cell lines [59]. Inhibition of MEK/ERK pathway by BI-847325 increased the expression of pro-apoptotic gene *BIM* and decreased anti-apoptotic genes *NFκB1* and *NFκB2* as well as *AURKA* and *AURKB* in ATC cells (Fig. 1). The treatment with BI-847325, a dual MEK/Aurora kinase inhibitor, also induced apoptosis at the cellular level (Fig. 2E–H) by decreasing the function of AURKA, AURKB, and AURKC in addition to regulating the expression of apoptotic genes. Phadke et al. showed that BI-847325 treatment regulated the expression of Mcl-1 and BIM and consequently induced apoptosis in melanoma cell lines [60].

Moreover, in our previous study, we determined the effect of BI-847325 anticancer drug on the molecular mechanisms that regulate MALAT1-related genes in ATC. The results of this study suggested that BI-847325 could be effective against ATC by regulating the genes involved in cell cycle and apoptosis including MALAT1 and its downstream genes such as *Mcl-1*, *miR-363-3p*, and *cyclin D1* [22].

Evidence has shown that RAS/RAF/MEK/ERK pathway and Aurora kinase family are also involved in cell cycle and proliferation [5, 9]. Activation of the MAPK signaling pathway as a result of mutations occurring in the *RAS* and *BRAF* genes can lead to rapid tumor progression and poor prognosis of ATC [61]. Over the past decade, our knowledge about the molecular details that control cell cycle and proliferation has improved. Hence, it has been shown that different genes such as *P16*, *AURKA*, and *AURKB* play an important role in tumor growth in ATC [9, 62–64]. Lee et al. demonstrated that the P16 tumor suppressor, which inhibits cell cycle progression, is undetectable in 24 of 27 ATC tissues [65, 66]. Moreover, Wiseman et al. using immunohistochemistry (IHC) and tissue microarray (TMA) found that the AURKC is one of the five molecular targets most frequently expressed in 32 ATC patients’ tissue samples [67]. Baldini et al. showed that selective AURKA and AURKB inhibitors have therapeutic potential for ATC due to suppression of cell proliferation as well as induction of apoptosis and G2/M phase arrest [10]. Chromatin compaction in G2/M is mediated by Histone H3 phosphorylation. AURKB promotes mitosis and cell division by increasing the phosphorylation of Histone H3 in serine 10 [68] while AURKB inhibitors decrease cell proliferation by reducing this phosphorylation [69]. Consistent with the findings of Phadke et al. [60], the results of this study showed that the treatment with BI-847325 could reduce phosphorylation of Histone H3 in serine 10 in ATC cell lines (Fig. 4D). Also, Sorrentino et al. showed that AURKB overexpressed in ATC cells and its inhibition by RNA interference or AURK inhibitor can reduce cell growth [70]. Because the expressions of *P16*, *AURKA*, and *AURKB* genes are regulated by the MAPK pathway [54, 55, 71], BI-847325 as a dual MEK/Aurora kinase inhibitor significantly increased *P16* expression in both ATC cell lines. This anticancer drug also decreased the expression and function of key regulators of cell cycle and proliferation, AURKA, AURKB, and AURKC, resulting in cell cycle arrest and reduced cell proliferation (Fig. 2A–D and I–K). It should be noted that the ATC cell lines used in this study have mutations in the MAPK pathway i.e. *RAS* mutation [C643] and *BRAF*^V600E^ mutation [SW1736] [20] and, therefore their different responses to BI-847325 at the cellular and molecular levels may be related to their different genetic backgrounds.

Overall, BI-847325 can be suggested as an anticancer drug for the treatment of ATC patients due to its regulatory effects on key biological processes associated with cancer progression.

## Conclusion

In conclusion, the results of this study showed that BI-857,325 has the potential to be considered as an effective multi-targeted anticancer drug for the treatment of ATC. BI-847325 intervention induced the thyroid-specific differentiation markers including NIS and Tg as well as apoptosis in ATC cell lines. Moreover, targeted therapy with BI-847325 reduced invasion, angiogenesis, proliferation, cell cycle progression, and multidrug resistance at the molecular and/or cellular levels. In addition, BI-847325 decreased the function of MEK1/2 and AURKB by decreasing the levels of phospho-ERK1/2 and phospho-Histone H3. However, further experiments using xenograft transplant tumors and ATC animal models are needed to confirm the findings of this study on the response of ATC tumors to BI-847325 anticancer drug in vivo. Also, the use of additional complementary assays to determine the details of the regulatory mechanisms of this hybrid compound on the biological processes involved in cancer progression can help to better understand the effects of BI-847325.

.

## Supplementary information

**Additional file 1: Figure S1.** Uncropped immunoblots on X-ray film and PVDF membrane for the western blot bands are presented in Fig. 4 (D). The molecular weight marker was loaded on each gel. All samples were run into 2 gels, one for β-actin and the other one for the rest of the antibodies (4 antibodies). Following immunoblotting, the blot used for 4 antibodies was cut into 4 parts, 2 upper parts were probed with ERK1/2 and phospho-ERK1/2 antibodies, and 2 lower parts were probed with Histone H3 and phospho-Histone H3 antibodies. The molecular weight markers are not shown on some of the cut blots.

## Data Availability

The datasets generated and/or analyzed during the current study are available from the corresponding author upon reasonable request.

## Abbreviations

2D: Two-dimensional
3D: Three-dimensional
ATC: Anaplastic Thyroid Cancer
AURKA: Aurora Kinase A
AURKB: Aurora Kinase B
AURKC: Aurora Kinase C
BIM: BCL2 Like 11
IC_50_: Half-Maximal Inhibitory Concentration
IHC: Immunohistochemistry
IRMA: Immunoradiometric assay
MALAT1: Metastasis Associated Lung Adenocarcinoma Transcript 1
MAPK: Mitogen-Activated protein kinase
Mcl-1: Myeloid cell leukemia sequence 1
MDR1: ATP Binding Cassette Subfamily B Member 1
MRP1: ATP Binding Cassette Subfamily C Member 1
NF*κ*B1: Nuclear Factor Kappa B Subunit 1
NF*κ*B2: Nuclear Factor Kappa B Subunit 2
NIS: Sodium/Iodide Symporter
P16: Cyclin Dependent Kinase Inhibitor 2 A
qRT-PCR: quantitative Real-Time Polymerase Chain Reaction
Tg: Thyroglobulin
TMA: Tissue microarray
VEGF: Vascular endothelial growth factor
