# Supplementary material for: BI-847325, a selective dual MEK and Aurora kinases inhibitor, reduces aggressive behavior of anaplastic thyroid carcinoma on an in vitro three-dimensional culture
Source: Cancer Cell Int. 2022 Dec 8;22:388. doi: 10.1186/s12935-022-02813-6 (PMC9730667; doi:10.1186/s12935-022-02813-6)
Supplement: Supplementary file 1 — Additional file 1: Figure S1. Uncropped immunoblots on X-ray film and PVDF membrane for the western blot bands are presented in Fig. 4 (D). The molecular weight marker was loaded on each gel. All samples were run into 2 gels, one for β-actin and the other one for the rest of the antibodies (4 antibodies). Following immunoblotting, the blot used for 4 antibodies was cut into 4 parts, 2 upper parts were probed with ERK1/2 and phospho-ERK1/2 antibodies, and 2 lower parts were probed with Histone H3 and phospho-Histone H3 antibodies. The molecular weight markers are not shown on some of the cut blots. [file 12935_2022_2813_MOESM1_ESM.pdf]

Supplementary Information

BI-847325, a Selective Dual MEK and Aurora Kinases Inhibitor, Reduces Aggressive Behavior of Anaplastic Thyroid Carcinoma on an in vitro Three-Dimensional Culture

Hilda Samimi[1,2], Rezvan Tavakoli[3], Parviz Fallah[4], Alireza Naderi Sohi[1], Maryam Amini Shirkouhi[1], Mahmood Naderi[5], Vahid Haghpanah[6,1*]

[1] Endocrinology and Metabolism Research Center, Endocrinology and Metabolism Clinical Sciences Institute, Tehran University of Medical Sciences, Tehran, Iran

[2] Department of Biology, Science and Research Branch, Islamic Azad University, Tehran, Iran

[3] Hepatitis and HIV Department, Pasteur Institute of Iran, Tehran, Iran

[4] Department of Laboratory Science, Faculty of Allied Medicine, Alborz University of Medical Sciences, Karaj, Iran

[5] Digestive Diseases Research Center, Digestive Diseases Research Institute, Tehran University of Medical Sciences, Tehran, Iran

[6] Personalized Medicine Research Center, Endocrinology and Metabolism Clinical Sciences Institute, Tehran University of Medical Sciences, Tehran, Iran

*Corresponding author:

Vahid Haghpanah, MD MPH PhD
Endocrinology and Metabolism Clinical Sciences Institute
Tel: +98 21 88220037-8
Fax: +98 21 88220052
Email: v.haghpanah@gmail.com
vhaghpanah@tums.ac.ir

X-ray film	X-ray film + PVDF membrane

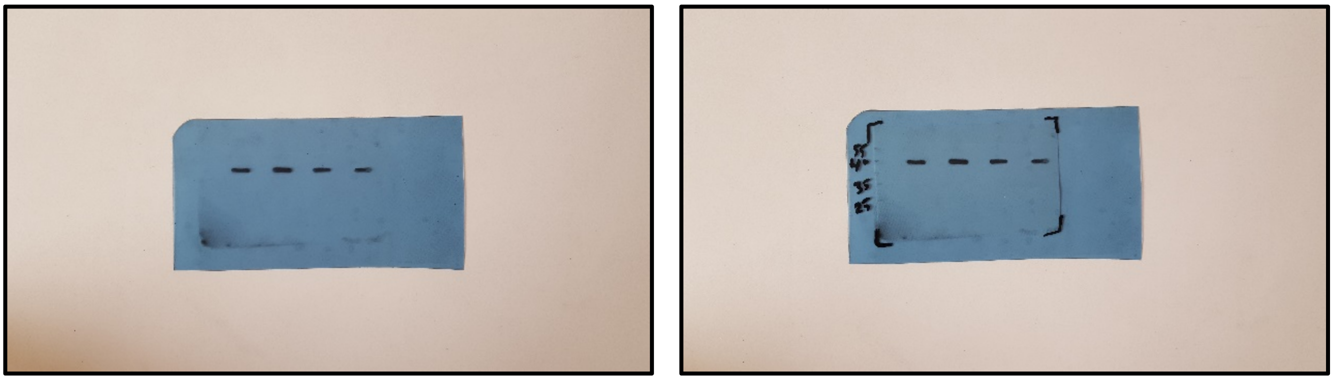

β-actin, 42 kDa

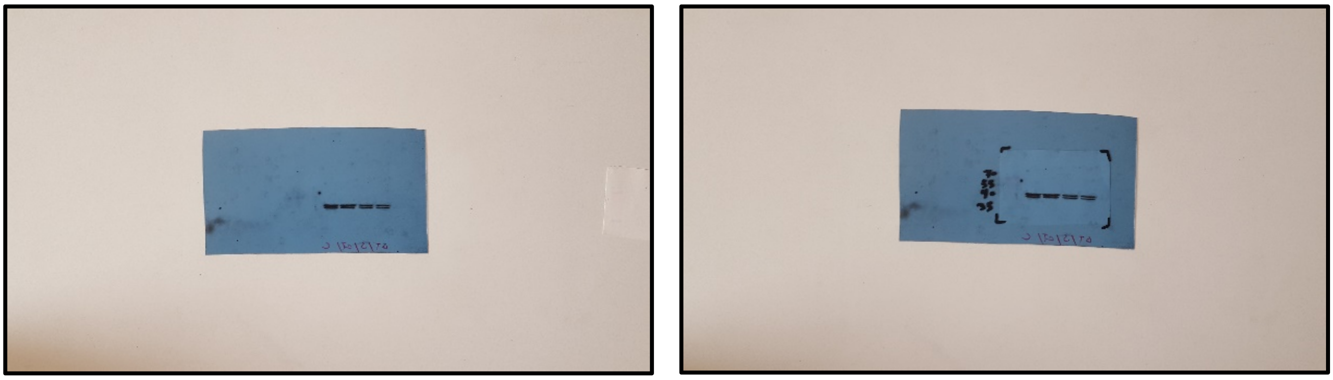

ERK1/2, 44/42 kDa

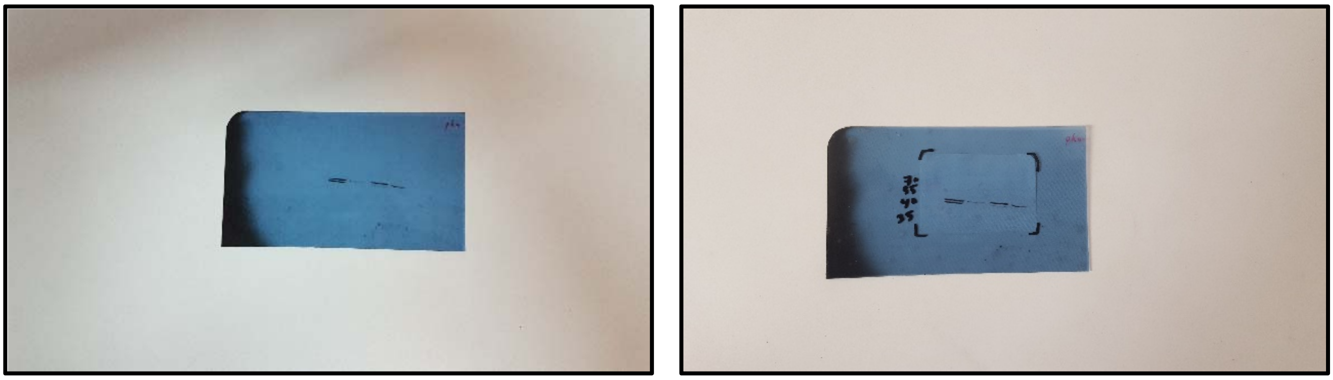

phospho-ERK1/2, 44/42 kDa

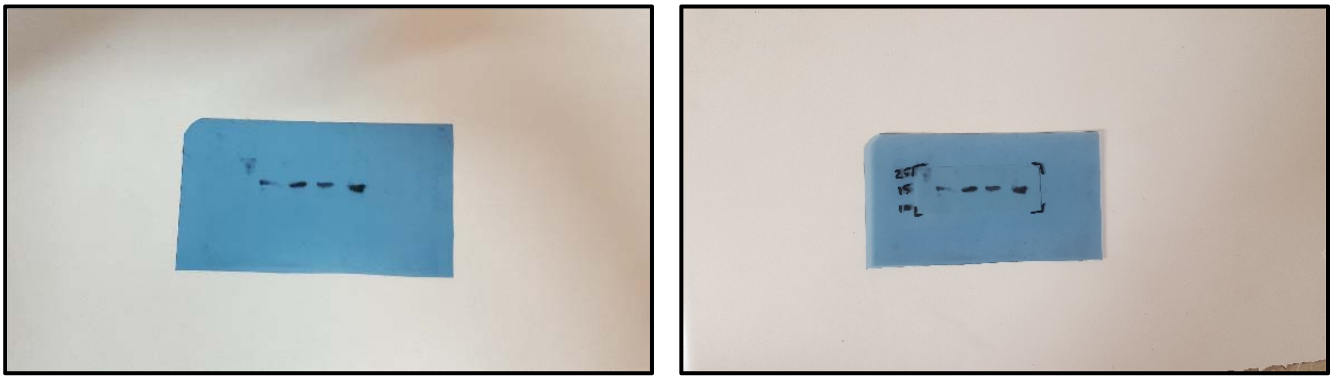

Histone H3, 17 kDa

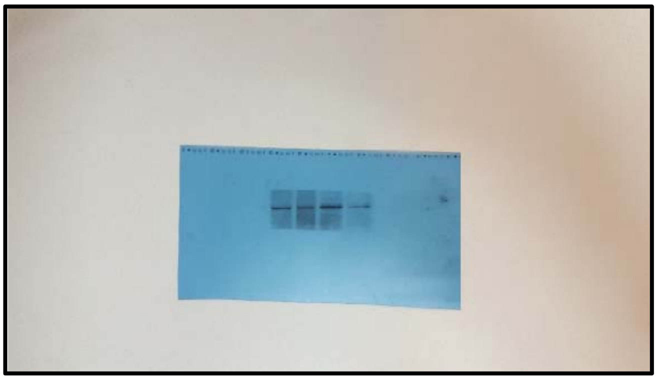 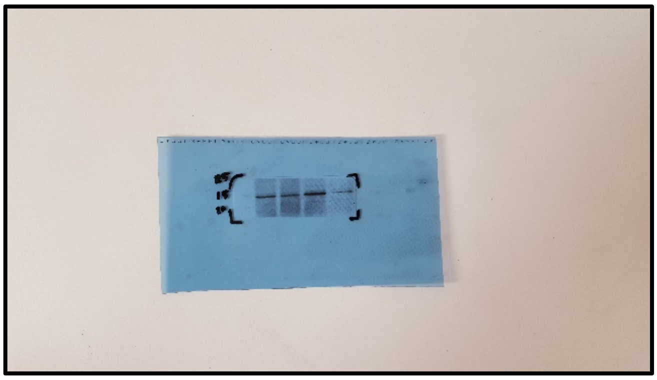

phospho-Histone H3, 17 kDa

Supplementary Figure S1. Uncropped immunoblots on X-ray film and PVDF membrane for the western blot bands are presented in Figure 4 (D). The molecular weight marker was loaded on each gel. All samples were run into 2 gels, one for β-actin and the other one for the rest of the antibodies (4 antibodies). Following immunoblotting, the blot used for 4 antibodies was cut into 4 parts, 2 upper parts were probed with ERK1/2 and phospho-ERK1/2 antibodies, and 2 lower parts were probed with Histone H3 and phospho-Histone H3 antibodies. The molecular weight markers are not shown on some of the cut blots.

*The order of samples is similar to Figure 4 (D).
